# Long non-coding RNA as a potential biomarker for prognosis of glioma: A protocol for systematic review and meta-analysis

**DOI:** 10.1097/MD.0000000000026921

**Published:** 2021-08-20

**Authors:** Teng Xie, Bin Li, Huaming Liu, Chunwei Zhang, Yanhua Wang, Zhijun Chen, Junping Yan

**Affiliations:** a Department of Neurosurgery, The People's Hospital of Hanchuan, Hanchuan, Hubei province, China.; b Department of Neurosurgery, The First People's Hospital of Jingmen, Jingmen, Hubei province, China.

**Keywords:** glioma, long non-coding RNA, meta-analysis, prognosis, protocol

## Abstract

**Background:**

The molecular mechanism of Glioma is still unclear, and there are few early diagnostic markers. Therefore, it is urgent to figure out effective preventive measures, active diagnostic methods and rapid treatment measures. In recent years, relevant studies have revealed that long non-coding RNA (lncRNA) is associated with the prognosis of Glioma. However, these results have not been supported by any evidence. Therefore, this study carried out a meta-analysis method to analyze the relationship between lncRNA and the prognosis of Glioma. In addition, bioinformatics analysis was conducted to investigate the mechanism and related pathways of lncRNAs in Glioma.

**Methods:**

We performed a systematic search in electronic databases, including China National Knowledge Infrastructure, Chinese Biomedical literature Database, Chinese Scientific and Journal Database, Wan Fang database, PubMed, EMBASE, Cochrane Library and Web of Science, to investigate the potential association between lncRNA expression and prognostic significance and clinical features in glioma patients. Hazards ratios (HRs) with corresponding 95% confidence intervals (CIs) were pooled to estimate the prognosis value of lncRNA by Stata16.0 software. The online tool AnnoLnc was applied to screen the co-expressed gene related to each lncRNA, David was used for gene ontology (GO) analysis and enrichment analysis of the signal pathway, and through Starbase, the possible competitive endogenous RNA network of lncRNAs was constructed.

**Results:**

The results of this meta-analysis would be submitted to peer-reviewed journals for publication.

**Conclusion:**

This study will provide evidence-based medical evidence for lncRNA, so as to predict the prognosis of Glioma and bioinformatics analysis will provide ideas for the mechanism study on Glioma.

## 1 Introduction

Human glioma is a clinically common intracranial tumor, and the average survival time of patients with malignant glioma after diagnosis is less than 1 year.^[1]^ In recent years, surgery, chemoradiotherapy, targeted therapy, and biologic therapy have all developed. However, there was no significant improvement in the prognosis of patients with glioma.^[2]^ Therefore, it is very important to explore the underlying molecular mechanism of the occurrence and development of glioma to find effective diagnostic and therapeutic targets.

Although the molecular mechanism, genetic mechanism and related pathways of glioma have been widely explored, the exact pathogenesis of glioma has not been clarified yet.^[3]^ Long non-coding RNA (lncRNA) is a class of non-coding RNAs with a length of more than 200 bases. Although it does not participate in protein coding, many recent studies have proved that lncRNA is involved in various life activities in vivo, such as the diversity of embryonic stem cells, the regulation of cell cycle, the occurrence and development of cancer, etc.^[4]^ Nowadays, more and more studies have confirmed that lncRNAs can be used as diagnostic markers of cancer, and even as a treatment option.^[5]^ Recent studies have illustrated that the abnormal expression of some lncRNAs is closely related to the recurrence and clinical prognosis of gliomas, and it can be used as potential biomarkers, prognostic indicators and even therapeutic targets of gliomas.^[6–8]^

Although many studies have indicated that lncRNA may be a potential prognostic biomarker for glioma, these studies have limitations such as small sample size and discrete data.^[6,9–12]^ In this study, systematic review and meta-analysis were carried out to evaluate the relationship between lncRNA expression and prognosis of glioma patients. In addition, this study constructed a competitive endogenous RNA network using bioinformatics technology to further explore its regulatory mechanism in glioma. It could also predict its potential application in the prognosis of glioma and provide new biomarkers for the diagnosis, treatment and prognosis of glioma.

## 2 Methods

### 2.1 Study registration

This meta-analysis protocol is based on the Preferred Reporting Items for Systematic Reviews and Meta-analysis Protocols (PRISMA-P) statement guidelines.^[13]^ The protocol of the systematic review was registered on Open Science Framework, and the registration number is DOI 10.17605/OSF.IO/BXMFK.

### 2.2 Data sources and retrieval strategy

We searched the China National Knowledge Infrastructure, Chinese Biomedical literature Database, Chinese Scientific and Journal Database, Wan Fang database, PubMed, EMBASE, Cochrane Library and Web of Science databases to identify all potentially eligible articles from inception to July 2021. The detailed search strategies are listed in Table 1.

**Table 1:** Search strategy in PubMed database.

#1 Glioma [MeSH]
#2 Glial Cell Tumors [Title/Abstract]
#3 Malignant Glioma [Title/Abstract]
#4 Mixed Glioma [Title/Abstract]
#5 Glial Cell Tumor [Title/Abstract]
#6 Glioma, Malignant [Title/Abstract]
#7 Glioma, Mixed [Title/Abstract]
#8 Gliomas [Title/Abstract]
#9 Gliomas, Malignant [Title/Abstract]
#10 Gliomas, Mixed [Title/Abstract]
#11 Malignant Gliomas [Title/Abstract]
#12 Mixed Gliomas [Title/Abstract]
#13 Tumor, Glial Cell [Title/Abstract]
#14 Tumors, Glial Cell [Title/Abstract]
#15 or/1–4
#16 RNA, Long Untranslated [MeSH]
#17 LINC RNA [Title/Abstract]
#18 LincRNAs [Title/Abstract]
#19 Long Intergenic Non-Protein Coding RNA [Title/Abstract]
#20 Long Non-Coding RNA [Title/Abstract]
#21 Long Non-Protein-Coding RNA [Title/Abstract]
#22 Long Noncoding RNA [Title/Abstract]
#23 Long ncRNA [Title/Abstract]
#24 Long ncRNAs [Title/Abstract]
#25 RNA, Long Non-Translated [Title/Abstract]
#26 Long Intergenic Non Protein Coding RNA [Title/Abstract]
#27 Long Non Coding RNA [Title/Abstract]
#28 Long Non Protein Coding RNA [Title/Abstract]
#29 Long Non-Translated RNA [Title/Abstract]
#30 Long Untranslated RNA [Title/Abstract]
#31 Non-Coding RNA, Long [Title/Abstract]
#32 Non-Protein-Coding RNA, Long [Title/Abstract]
#33 Non-Translated RNA, Long [Title/Abstract]
#34 Noncoding RNA, Long [Title/Abstract]
#35 RNA, Long Non Translated [Title/Abstract]
#36 RNA, Long Non-Coding [Title/Abstract]
#37 RNA, Long Non-Protein-Coding [Title/Abstract]
#38 RNA, Long Noncoding [Title/Abstract]
#39 Untranslated RNA, Long [Title/Abstract]
#40 ncRNA, Long [Title/Abstract]
#41 ncRNAs, Long [Title/Abstract]
#42 or/16–41
#43 Prognos^∗^ [Title/Abstract]
#44 Overall survival [Title/Abstract]
#45 Progression-free survival [Title/Abstract]
#46 Recurrence-free survival [Title/Abstract]
#47 Hazards ratio [Title/Abstract]
#48 Disease-free survival [Title/Abstract]
#49 Survival [Title/Abstract]
#50 or/43–49
#51 #15 and #42 and #50

### 2.3 Inclusion criteria for study selection

#### 2.3.1 Inclusion criteria

1. Studies on human glioma;
2. Expression of lncRNA in the tissue specimens of patients with glioma were detected by established molecular methods;
3. The relationship between the expression of lncRNA and the prognosis of patients with glioma was analyzed. The prognostic endpoints included overall survival (OS), progression-free survival (PFS), recurrence-free survival (RFS), and disease-free survival (DFS);
4. Hazard ratios (HRs) and 95% confidence intervals (CIs) were directly extracted from the univariate or multivariate analysis, or with the application of Engauge Digitizer4.1 to convert Kaplan-Meier survival curves.

#### 2.3.2 Exclusion criteria

1. Repeatedly published research;
2. Preclinical in vitro or in vivo experimental studies were excluded;
3. Case reports, letters, expert opinions, meeting records, review articles, commentaries, and clinical guidelines were excluded;
4. Studies without HRs or 95% CIs were excluded.

### 2.4 Data collection and analysis

**Figure 1:**
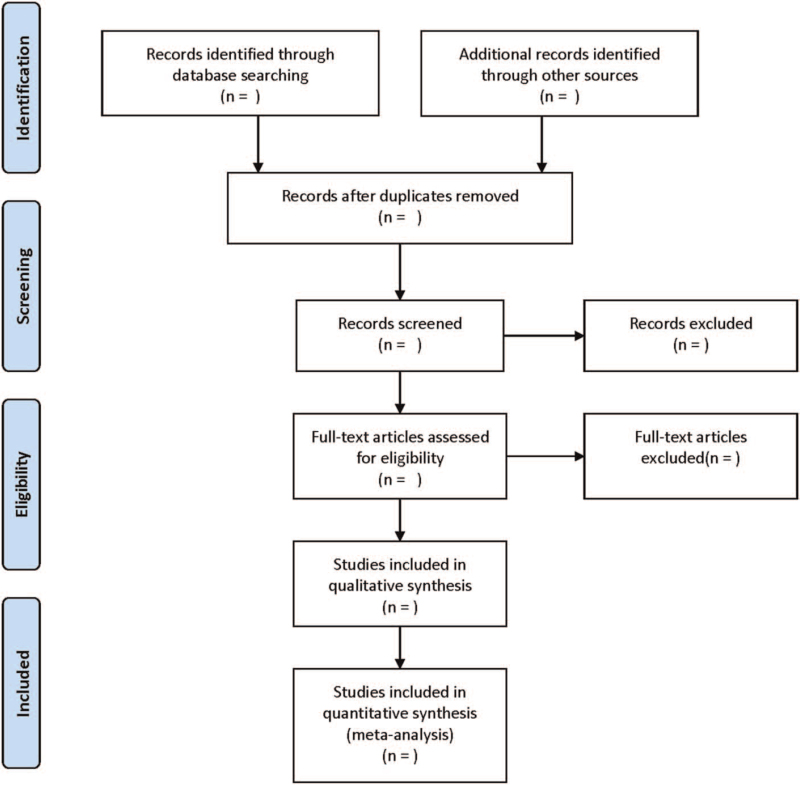
Flow diagram of study selection process.

Databases were searched and the publication was independently evaluated by two institutes. The included studies were selected through consensus. The following data were obtained from each eligible study: First author, year of publication, ethnicity, country, number of patients, assay for lncRNA expression, cut-off value, OS, PFS, RFS, and DFS. The HRs and 95%CIs were extracted directly from univariate or multivariate analyses, or Kaplan–Meier survival curves were converted with Engauge Digitizer4.1. The literature screening process is displayed in Figure 1.

### 2.5 Quality assessment

The quality of each study was assessed using the Newcastle–-Ottawa Scale (NOS) and was quantitatively evaluated by two independent reviewers.^[14]^ The scores for quality assessment ranged from 0–9, and studies with a NOS score >6 were considered to be high quality.^[15]^

### 2.6 Measures of prognosis

OS, PFS, RFS, and DFS were taken as prognostic outcomes, and the results were expressed as HRs with 95% CIs.

### 2.7 Management of missing data

If there exists insufficient or missing data in the literature, we would only analyze the currently available data and discuss the potential value.

### 2.8 Statistical analysis

Meta-analysis was conducted on the Stata 16.0 (Stata Corporation, TX). HR and its 95% CIs were applied to evaluate the relationship between lncRNA expression and prognosis in patients with glioma. Heterogeneity was tested by Q-statistic and I^2^-statistic, I^2^ > 50% was considered as significant heterogeneity, and the random-effects model or the fixed-effects model was adopted. The *P* values in this study were two-sided, and *P* < .05 indicated that there were statistical significances.

### 2.9 Additional analysis

#### 2.9.1 Subgroup analysis

According to the detection methods of LncRNA, ethnicity, and the up-regulation and down-regulation of lncRNA, we analyzed the subgroup.

#### 2.9.2 Sensitivity analysis

Sensitivity analysis was performed via sequential deletion of a single included study to test.

#### 2.9.3 Reporting bias

The publication bias was evaluated by performing Begg's test and Egger's test.^[16,17]^

## 3 Bioinformatics analysis

### 3.1 Screening of genes related to lncRNAs

Genes that might be related to lncRNA expression level were screened by the online tool AnnoLnc (http://annolnc.cbi.pku.edu.cn),^[18]^ and the intensity of co-expression was indicated by the interaction score.

### 3.2 GO analysis and signal pathway analysis

The online tool David (https://david.ncifcrf.gov/) was used to conduct GO analysis and signal pathway analysis on the screened co-expressed genes.

### 3.3 Construction of competitive endogenous RNA networks

The online tool Starbase (http://starbase.sysu.edu.cn/starbase2/) was used to study miRNA molecules targeted by lncRNAs and downstream target mRNA molecules. The online database GEPIA (http://gepia.cancer-pku.cn/) was applied to explore the expression level and survival of target mRNA molecules in glioma.

## 4 Ethics

Our research data were derived from published literatures, because there were no patient recruitment and personal information collection. Therefore, ethical approval was not required.

## 5 Discussion

LncRNA plays an important biological role in human diseases.^[19]^ Abnormal expression of lncRNAs may potentially alter basic cellular biological processes and contribute to tumor genesis.^[20]^ Studies have exhibited that lncRNA may be a prognostic factor and therapeutic target in patients with glioma.^[21,22]^ Given the limited sample size, the results of a single study are not convincing. Therefore, we conducted a meta-analysis to explore the effects of lncRNA expression on the prognosis of glioma. In addition, the construction of competitive endogenous RNA networks by bioinformatics will reveal the genesis and development of glioma, and provide a basis for the exploration on the molecular mechanisms in the future.

## Author contributions

**Conceptualization:** Junping Yan, Teng Xie.

**Data curation:** Bin Li.

**Formal analysis:** Bin Li.

**Funding acquisition:** Junping Yan.

**Investigation:** Bin Li.

**Methodology:** Bin Li.

**Project administration:** Junping Yan.

**Resources:** Huaming Liu, Chunwei Zhang.

**Software:** Huaming Liu, Chunwei Zhang.

**Supervision:** Junping Yan.

**Validation:** Chunwei Zhang, Yanhua Wang, Zhijun Chen.

**Visualization:** Yanhua Wang, Zhijun Chen.

**Writing – original draft:** Junping Yan, Teng Xie.

**Writing – review & editing:** Junping Yan, Teng Xie.
